# A model-based estimation of inter-prefectural migration of physicians within Japan and associated factors: A 20-year retrospective study

**DOI:** 10.1097/MD.0000000000010878

**Published:** 2018-06-01

**Authors:** Naoki Okada, Tetsuya Tanimoto, Tomohiro Morita, Asaka Higuchi, Izumi Yoshida, Kazuhiro Kosugi, Yuto Maeda, Yoshitaka Nishikawa, Akihiko Ozaki, Kenji Tsuda, Jinichi Mori, Mutsuko Ohnishi, Larry Wesley Ward, Hiroto Narimatsu, Koichiro Yuji, Masahiro Kami

**Affiliations:** a Keio University; b Navitas Clinic, Tokyo; c Jyoban Hospital of Tokiwa Foundation; d Soma Central Hospital, Fukushima; e Medical Governance Research Institute, Tokyo; f National Cancer Center Hospital East, Chiba; g National Center for Child Health and Development, Tokyo; h Kyoto University, Kyoto; i Ohmachi Hospital, Fukushima; j Graduate School of Public Health, Teikyo University, Tokyo; k Kanagawa Cancer Center, Kanagawa; l Institute of Medical Sciences, University of Tokyo, Tokyo, Japan.

**Keywords:** inequality, Japan, migration, misdistribution, physicians

## Abstract

Despite an increase in the number of physicians in Japan, misdistribution of physicians within the 47 prefectures remains a major issue. Migration of physicians among prefectures might partly explain the misdistribution. However, geographical differences and the magnitude of physicians’ migration are unclear. The aim of this study was to estimate the extent of migration of physicians among prefectures and explore possible factors associated with physicians’ migration patterns.

Using a publicly available government database from 1995 to 2014, a quantitative estimation of physicians’ migration after graduation from a medical school was performed. The inflow and outflow of physicians were ostensibly calculated in each prefecture based on the differences between the number of newly licensed physicians and the actual number of practicing physicians after an adjustment for the number of deceased or retired physicians. Simple and multiple linear regression analyses were conducted to examine socio-demographic background factors.

During the 20-year study period, the mean annual numbers of newly licensed physicians, deceased or retired physicians, and increase in practicing physicians in the whole country were 7416, 3382, and 4034, respectively. Among the 47 prefectures, the median annual number of newly licensed physicians to 100,000 population ratio (PPR) was 6.4 (range 1.5–16.5), the median annual adjusted number of newly licensed physicians was 61 (range, −18 to 845; the negative and positive values denote outflow and inflow, respectively), whereas the median annual number of migrating physicians was 13 (range, −171 to 241). The minimum and maximum migration ratios observed were −68% and 245%, respectively. In the final regression model of the 8 variables examined, only “newly licensed PPR” remained significantly associated with physician's migration ratios.

A significant inequality in the proportion of the migration of physicians among prefectures in Japan was observed. The multivariate analyses suggest that the newly licensed PPRs, and not from-rural-to-urban migration, might be one of the keys to explaining the migration ratios of physicians. The differences and magnitude of physicians’ migration should be factored into mitigate misdistribution of physicians.

## Abstract 2

Supplemental Digital Content is available in the text

## 1 Introduction

To achieve an adequate supply of health professionals is one of the most challenging issues all over the world.^[1,2]^ Misdistribution of physicians due to international migration among low-, middle- and high-income countries is an area of concern. This is also true for domestic migration from rural to urban areas.^[3–9]^ Japan is categorized as one of the high-income countries and suffers from an undersupply of physicians in some of its 47 prefectures.^[8]^ With a relatively large population of approximately 127 million in 2016 within a wide variety of geographic areas of approximately 378,000 km^2^,^[10]^ each prefecture has its own characteristic background. Hence, public health policy makers in the government struggle with devising methods to meet the various public demands for better health care delivery.^[7–9]^

Furthermore, in Japan, the aging population has been increasing rapidly. The number of people in Japan aged 65 years or older steadily grew from just 4.9% in 1950 to 7.1% in 1970, 12.1% in 1990, and 25% in 2013.^[10]^ The increase in the aging population has led to a significant increase in the physicians’ workload. Our previous study predicted that physician shortages may exacerbate by 2035, causing a significant problem in the country.^[8]^ Historically, the number of physicians in Japan has been increasing over the past 50 years.^[11]^ In 1961, when universal health care coverage was established,^[12]^ there were 46 medical schools, with an annual admission quota of 2840 students. In addition, the country had about 100,000 physicians representing 103.6 physicians per 100,000 population ratio (PPR). With 16 prefectures out of 47 left without a medical school at that time, there were pressing demands to increase the number of physicians to counter the inequality of medical resources among prefectures.

During the 1970s, new medical schools were established in succession under the initiative of the government (under the slogan of “at least one medical school in each prefecture”) to alleviate the problem of physician shortage.^[6]^ The number of medical schools increased to 80 by 1981, and the annual admission quota increased up to 8280. After concerns over physician surplus emerged in 1985, the government started to decrease the admission quota to 7625 by 2007. During that period, the rapidly aging population and advanced medical technology led to concerns about the inequality and undersupply of medical resources.^[8]^ In 2008, the government began increasing the admission quota, which reached up to 9262 by 2016.^[13,14]^ In 2016, the country had 319,480 physicians.^[11]^ The government also decided to establish 2 new medical universities, one of which opened in 2016 and one in 2017.^[13]^

Although the total number of physicians in the whole country has been increasing gradually as mentioned earlier and the lack of physicians has been mitigated, the inter-prefectural inequality within the country still remains as a significant problem.^[7–9,15]^ In 2016, the practicing PPR (ie, the number of practicing physicians in a clinic or a hospital, excluding those who engage in non-clinical research, education, government administration, and those who have retired) was 240.1 throughout the country. Among all 47 prefectures, the 3 highest practicing PPRs were 315.9 in Tokushima, 314.9 in Kyoto, and 306.0 in Kochi. On the contrary, the 3 lowest ratios were 160.1 in Saitama, 180.4 in Ibaraki, and 189.9 in Chiba.^[11,16]^ Under the circumstance, although political debates regarding the causes of inequalities continue with an abundance of preconception and a paucity of evidence, some advocates believe that a mitigation policy, such as obligatory service in rural areas, should be implemented to reduce the inequality of physicians’ distribution among prefectures.

However, the migration of physicians within the country has not been extensively investigated, unlike international migration between low- and middle-income countries and high-income countries.^[1,17–20]^ The migration patterns within countries such as Canada and the United States have been assessed.^[3–5]^ In Japan, a previous study reported the migration pattern of physicians in only one prefecture.^[21]^ Another study analyzed the retention of graduates within the vicinity of medical schools.^[22]^ Most Japanese physicians can choose their workplaces irrespective of the medical school they graduated from. There is no thoroughly planned or mandatory disposition of physicians in each prefecture, except for a minor proportion of newly licensed physicians who have to perform obligatory service because they availed a governmental subsidy.^[23,24]^ Therefore, the current distribution of physicians would mostly reflect the cumulative decisions that physicians make about their workplaces after graduation. However, without an official tracking system for noting their workplaces after graduation, there has been limited quantitative data on the number of physicians who choose to remain in the prefecture of their alma mater or migrate to another prefecture.

The aim of this study was to estimate the extent of migration of physicians among prefectures and explore possible factors associated with physicians’ migration patterns, although we could not obtain detailed personal reasons why physicians chose their workplace. Using publicly available data from the Japanese government, a model was constructed to ostensibly estimate the migration of physicians among prefectures through a 20-year period. In addition, publicly available background socio-demographic data were analyzed to explore potential influences on migration.

## 2 Methods

### 2.1 Data source

Utilizing the Japanese public database from the Ministry of Health, Labor and Welfare (MHLW), the number of Japanese physicians from all specialties was obtained for 20 years from 1995 through 2014 (this represents the longest available data at the time of analysis in October 2016).^[16]^ The updated data up to 2016 became available in December 2017.^[11]^ The MHLW conducts the Survey of Physicians, Dentists, and Pharmacists every 2 years to monitor workplaces of professionals and other basic demographic data. Personal data, such as their alma mater or salaries, are unavailable. The anonymized results and summary data on the numbers of practicing doctors in each prefecture are available on their website.^[16]^

The MHLW discloses yearly qualifying examination data of the Japanese National Medical Practitioners. From the database of Statistics Bureau of Japan, 2013 and 2014, background socio-demographic factors were obtained for each prefecture.^[10]^ This data also included population, population density, ratio of elderly population (65 years old or older), average income of the general population, unemployment ratio of the general population, and the number of medical graduates.

### 2.2 Newly licensed physicians

Newly licensed physicians, except those receiving a special scholarship from the government, can choose their workplaces at their discretion. However, 3 medical schools out of 80 deploy their graduates evenly to each prefecture or their specific course to fulfill public services: Jichi Medical University that services rural and remote areas, the National Defense Medical College that services the Self-Defense Forces, and the University of Occupational and Environmental Health that services industries. The graduates of those 3 universities were excluded from the current study, and only newly licensed physicians from 77 medical schools were included.

### 2.3 Deceased or retired physicians

**Figure 1:**
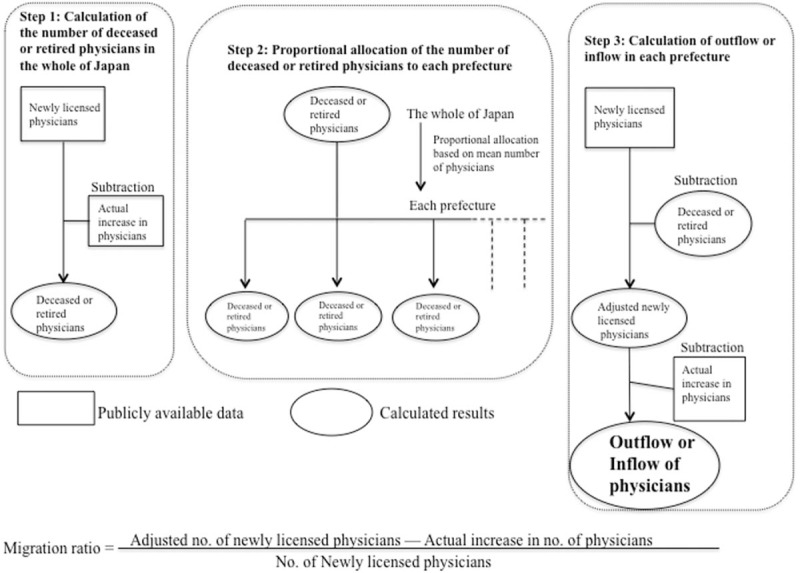
Methods used in calculating physicians’ migration. Since the official numbers of deceased or retired physicians in each prefecture are not available, we estimated the approximate numbers by using a calculation based on the publicly available numbers according to the 3 steps.

Since the official numbers of deceased or retired physicians in each prefecture are not available, estimates of the approximate numbers were performed as follows (Fig. 1). In Step 1, the increase in the number of physicians was calculated by subtracting the number of physicians in 1994 from that in 2014. The total number of deceased or retired physicians in the whole country was estimated by calculating the difference between the increase in the number of physicians and the numbers of newly licensed physicians. In Step 2, to estimate the number of deceased or retired physicians in each prefecture, the number in the whole country calculated in Step 1 was allocated to each prefecture in proportion to the mean numbers of physicians in that prefecture. The mean number of physicians over the 20-year period was calculated based on the database of the MHLW without considering the differences of age structure in each prefecture for approximation.

### 2.4 Estimates of migration ratios

The migration ratios of physicians in each prefecture were estimated as shown in Step 3 of Figure 1. The adjusted number of newly licensed physicians was calculated by subtracting the number of deceased or retired physicians from the number of newly licensed physicians in each prefecture. The adjusted number indicates the hypothetical increase in newly licensed physicians if there was no migration across the prefecture. Using the actual number of physicians obtained from the MHLW database, the number of physicians in each prefecture in 1994 was subtracted from that in 2014. This was defined as the actual increase in the number of physicians. When the adjusted number of newly licensed physicians was larger than the actual increase in the number of physicians, the prefecture was defined as an “outflow prefecture.” An “inflow prefecture” was defined as the opposite. Thus, a positive value of the calculated number of migrating physicians denotes outflow and a negative value denotes inflow. A migration ratio of inflow and outflow was estimated by dividing the inflow or outflow by the number of newly licensed physicians in each prefecture. Depending on the migration ratios, we divided the 47 prefectures into 4 groups (high outflow, N = 11; low outflow, N = 12; low inflow, N = 12; high inflow, N = 12).

### 2.5 Statistical analysis

The associations between the ratios of migration and each variable were examined using simple linear regression analyses. Subsequently, a multiple linear regression analysis was used to identify factors that were associated with physicians’ migration patterns. The ratio of migration (inflow and outflow) in each prefecture was used as a dependent variable. The following data were included in the analysis: the practicing PPR in 2014, newly licensed PPR, physician's average age, ratio of female physicians, population density in inhabitable land areas (assumed to be an indicator of urbanness and ruralness), unemployment ratio of the general population, ratio of aged population (65-years-old or older), and average income of the general population. All *P*-values <.05 were considered statistically significant. This study required no ethical approval because only publicly available data were used.

## 3 Results

During the 20-year study period, the mean annual numbers of newly licensed physicians, deceased or retired physicians, and the increase in the number of physicians in the whole country were 7416, 3382, and 4034, respectively. The median annual numbers of newly licensed physicians, deceased or retired physicians, and actual increase in physicians among all prefectures in the study period were 99 (range, 61–1285), 46 (range, 21–441), and 43 (range, 14–674), respectively. The median annual newly licensed PPR was 6.4 (range, 1.5–16.5). The median annual adjusted number of newly licensed physicians was 61 (range, −18 to 845), and the negative value indicates that the number of deceased or retired physicians surpassed the number of newly licensed physicians. When the actual increase in the number of physicians was subtracted from the adjusted number of newly licensed physicians, the median number of migrating physicians was −13 (range, −171 to 241). The negative and positive values denote inflow and outflow, respectively.

**Figure 2:**
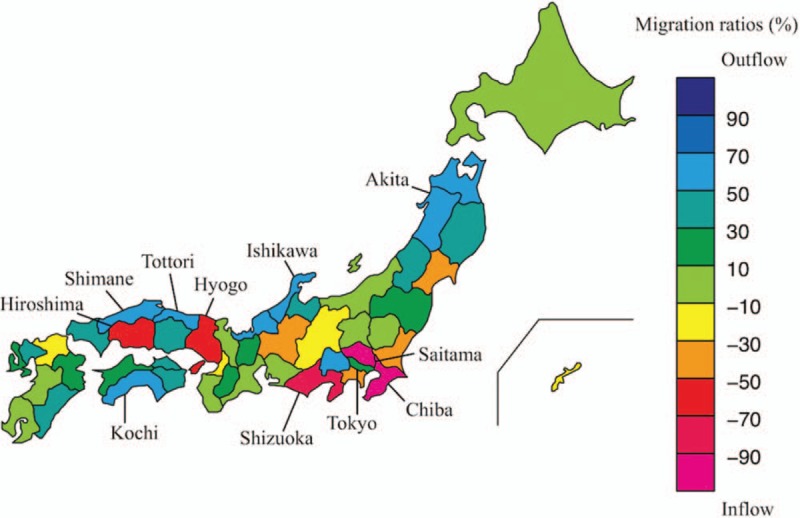
Migration ratios in each prefecture. The migration ratios are shown with color scales in each prefecture. The metropolis of Tokyo and the top 5 prefectures with the highest outflow and inflow ratios are indicated.

The migration ratios of physicians among all 47 prefectures and socio-demographic background factors are shown in Figure 2 and see Table, Supplemental content which illustrates the characteristics of the 47 prefectures. The maximum outflow and inflow ratio was 68% in Ishikawa prefecture and 245% in Chiba prefecture, with a maximum flow difference of 313%. Most of the outflow prefectures were in the rural regions facing the Sea of Japan or the Pacific Ocean (Ishikawa, Shimane, Kochi, Tottori, and Akita), while most of the inflow prefectures were peripheral zones of the metropolitan cities in more densely populated regions (Chiba, Saitama, Shizuoka, Hyogo, and Hiroshima). The metropolis of Tokyo, the most densely populated area, had an outflow ratio of 13%. Other urban prefectures, such as Aichi, Osaka, and Fukuoka, had modest inflow ratios, ranging from 7.7% to 22.8%.

Table 1 shows the 4 prefectural groups divided by the degree of migration ratios (ie, high and low outflow, and high and low inflow). The median migration ratios were 53.1% and 27.9% in high and low outflow groups, respectively; and 34.8% and −2.1% (ie, outflow) in the high and low inflow groups, respectively. Compared to the high inflow group, the high outflow group had a larger median practicing PPR (218.1 vs 250.9) and a larger median annual newly licensed PPR (3.7 vs 11.5). The high outflow group also had a larger median aged population ratio (23.8% vs 27.9%) and a higher average age of physicians (50.0 years vs 51.4 years). On the contrary, compared to the high inflow group, the high outflow group had a smaller median population density in inhabitable land areas (1564/m^2^ vs 646/m^2^), lower average income of the general population (JPY 3.22 million vs JPY 2.74 million), and a lower unemployment ratio of the general population (3.3% vs 2.8%).

**Table 1:**
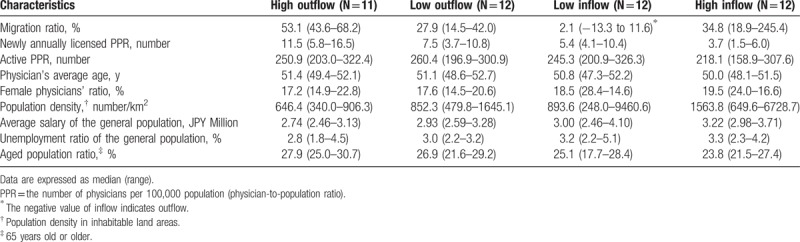
Characteristics of prefectures according to the migration ratios.

The results of the linear regression analyses are shown in Table 2. Simple linear regression analyses showed associations between physicians’ migration and five (including the practicing PPRs, the newly licensed PPRs, population density in inhabitable land areas, average income of the general population, and aged population ratio) of the 8 variables. However, after adjustment for these variables in the multiple regression analysis, only the newly licensed PPR was significantly associated with physicians’ migration (*P* < .001), that is, a high ratio led to increased outflow and a low ratio led to increased inflow.

**Table 2:**
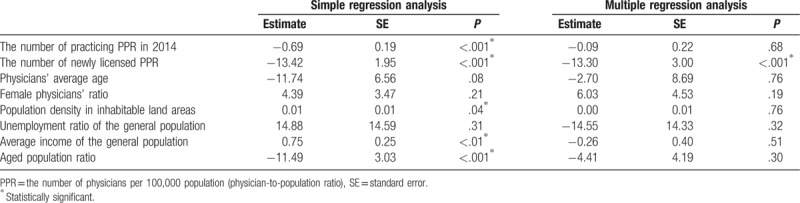
Simple and multiple linear regression analyses for physicians’ migration ratios.

## 4 Discussion

In this study, a model-based quantitative estimation of physicians’ migration after graduation was performed among 47 prefectures in Japan in a 20-year period. Significant differences among prefectures in the pattern of migration were revealed using the difference between the adjusted number of newly licensed physicians and the actual increase in the number of physicians as the numerator, and the number of newly licensed physicians as the denominator. When the numerator was positive, it was interpreted as an outflow of physicians, and when negative, it was interpreted as an inflow. In prefectures with high outflow, more than half of the newly licensed physicians ostensibly moved to another prefecture after graduation. On the contrary, in the prefectures with high inflow, more than twice the number of newly licensed physicians arrived from other prefectures. Since migration of physicians can be seen as a redistribution mechanism to influence inequality in the number of physicians throughout the country, recognizing and deciphering the pattern of migration would be useful in policy making to mitigate the misdistribution.

It should be noted that Japan has the relatively lower PPR compared to other high-income countries. In 2016, the country's practicing PPR was 240.1,^[11]^ while PPRs of Canada, the United Kingdom, and the United Stated ranged approximately from 260 to 280.^[2]^ PPRs of other European countries ranged as high as 300 to 500.^[1,2]^ Considering the advances in medical technology and the rapidly aging population, it would be reasonable to consider that a lack of physicians will continue in Japan in the future; although this has recently been mitigated by the increase in the admission quota.^[8]^ As our data indicated, the magnitude of physicians’ migration would have implications on the underlying mechanism concerning the development of unequal distribution of physicians which should be considered in policy decision making for underserved areas.

A trend, whereby physicians move from the rural to urban areas, appears to exist. While most urban prefectures, such as Aichi, Osaka, and Fukuoka, had modest inflow ratios, population density of inhabitable land areas (the indicator of urbanness and ruralness) was not identified as a significant factor for migration in our multivariate analysis. Notably, even the metropolis of Tokyo, which has 13 medical schools with an annual newly licensed PPR of 10.4 (9th among 47 prefectures), had an outflow ratio of 13%. The multivariate analyses suggest that the newly licensed PPRs, and not rural-urban migration, might be one of keys in explaining the migration ratios of physicians. However, migration of physicians may be driven by economic, social, and political variables that are peculiar to the local areas or jurisdictions where health professionals work. Under the Personal Data Protection Law of Japan, such data would be difficult to obtain for each physician, but further studies are warranted to determine the contributing factors related to migration.

Data also showed that specific urban prefectures neighboring Tokyo, such as Chiba and Saitama, had extremely high inflow ratios (Fig. 2 and Table, Supplemental Content which illustrates the characteristics of the 47 prefectures). Chiba and Saitama had only 1 medical school each despite their large population of 7.2 and 6.1 million, respectively. Therefore, high-school students were forced to choose a medical school located in another prefecture outside their own prefecture. It is plausible that after graduation, these students tended to choose a workplace near their hometown. In addition, because prefectures with high annual numbers of newly licensed physicians mostly had a high PPR, some physicians might have moved from more populated to underserved areas, and such migrations would have partly compensated for the inequality in PPR among prefectures.

A difference of more than 10 times in the newly licensed PPR among prefectures could be attributed to the inflexible medical education system. In the past, the government had established medical schools and their admission quota based on the prefectural divisions without fully considering the population and its chronological changes in each prefecture. Over the past decades, some urban prefectures experienced more than doubling of the population after the economic growth especially around the Great Metropolitan Tokyo areas.^[10]^ Other rural prefectures showed no change or experienced a decrease in their population.^[10]^ While some rural prefectures have had high admission quota per population that resulted in a high newly licensed PPR, other urban prefectures have been left with lower PPRs. The discrepancy between the healthcare workforce and the needs of the general public would be less if chronological geo-demographic factors had been taken into account within some prefectures. Therefore, the 2 previously mentioned newly established medical schools will play an important role in mitigating misdistribution in the country.

While specific background factors such as financial incentives, specialty choices and development, postgraduate training programs, and characteristic of towns could not be assessed, previous studies have shown that those factors were associated with a physician's choice of workplace.^[23,25–32]^ Since the medical system and social, cultural, economic, and political factors within each country vary, determinants of migration would vary depending on the context and personal factors in each country. To understand the underlying reasons for migration that are unique to Japan, more studies using novel methods are required. Migration of Japanese physicians has been scarcely monitored in the past. For medical students admitted into their institutions by regional quotas and a scholarship, the Japanese Council for Community-based Medical Education has recently begun geographic and specialty distributions until their retirement.^[33]^ The central government also plans to launch a tracking system of physicians’ workplace, and such data might help explain the underlying mechanism in migration pattern and be useful for policy making in the future.

Despite having an inflow ratio of >200%, Chiba and Saitama, 2 prefectures neighboring Tokyo, still had very low PPRs of 180.4 and 160.1, respectively, in 2016.^[16]^ Therefore, the inequality in PPR could not be sufficiently mitigated by migration alone. Consistent with this assumption, a previous Japanese study showed that an increase in the number of physicians between 1998 and 2008 did not lead to a more equal geographical distribution of physicians.^[34,35]^ Inequality remained even though the policy for increasing physicians mitigated the scarcity of physicians in medically underserved areas.^[7]^ Such findings are not fully recognized among the public because there have been a paucity of studies as well as discussions about the issue.

The government has spent a large amount of money on medical education, hence, Japanese physicians are generally considered by the public to make more effort to serve in the community healthcare. However, the absolute numbers of physicians are not high enough in some prefectures despite the relatively high newly licensed PPR (eg, Yamanashi prefecture has a PPR lower than the national average PPR of 230.2 and an outflow ratio of 52.9%, despite the high newly licensed PPR of 10.9). This illustrates the difficulty in mitigating the current misdistribution. If the inequality in PPR remains unsolved in the future, it might become a significant political agenda for the central as well as local governments. Nevertheless, it should be noted that health equality is distinct from health equity, and it might be better to set a minimum requirement for health outputs and/or health outcomes rather than pursue equality haphazardly. Regardless, the current study could contribute toward accelerating discussions within the Japanese government, and these findings might also be useful for policy making throughout global health communities.

### 4.1 Limitations

There are limitations to the current study. Due to the lack of data on the background factors that could be related to the reasons for migration, such as physicians’ salary, specialties, residency programs, family structure, and working styles; such factors were not studied. Furthermore, the multivariate linear regression analyses lacked the statistical power to detect significant factors with a small sample size of a total of 47 prefectures. Therefore, the reasons underlying the observed migration pattern still remain to be determined. Additionally, physicians who had gone abroad and international medical graduates who work in Japan were not included, although their number would be within allowance limits of error. The official numbers of deceased or retired physicians in each prefecture were not available; therefore, our calculations might include some errors. In addition, the number of physicians who graduated from Jichi Medical University, the National Defense Medical College, and the University of Occupational and Environmental Health were excluded from the analysis. Additionally, physicians who have received a special scholarship program to work in medically underserved areas were not considered.

## 5 Conclusion

A significant inequality in the proportion of migrating physicians among prefectures in Japan was observed in this 20-year study. The multivariate analyses suggest that the newly licensed PPRs, and not from-rural-to-urban migration, might be one of the keys to explaining the migration ratios of physicians. More information should be collected and disclosed to understand the reasons influencing the migration of physicians within Japan to mitigate the misdistribution of physicians.

## Author contributions

Conceived and designed the analyses: NO, TT, TM, and MK. All authors discussed the data and wrote the paper.

**Conceptualization:** Naoki Okada, Tetsuya Tanimoto, Masahiro Kami.

**Data curation:** Naoki Okada, Tomohiro Morita.

**Formal analysis:** Naoki Okada, Tetsuya Tanimoto, Tomohiro Morita, Hitoro Narimatsu.

**Funding acquisition:** Masahiro Kami.

**Investigation:** Naoki Okada, Tetsuya Tanimoto, Tomohiro Morita, Izumi Yoshida.

**Methodology:** Naoki Okada, Tetsuya Tanimoto.

**Project administration:** Tetsuya Tanimoto, Masahiro Kami.

**Software:** Tetsuya Tanimoto.

**Supervision:** Tetsuya Tanimoto.

**Validation:** Tetsuya Tanimoto, Tomohiro Morita.

**Visualization:** Tetsuya Tanimoto, Tomohiro Morita, Asaka Higuchi.

**Writing – original draft:** Tetsuya Tanimoto, Tomohiro Morita, Asaka Higuchi, Izumi Yoshida, Kazuhiro Kosugi, Yuto Maeda, Yoshitaka Nishikawa, Akihiko Ozaki, Kenji Tsuda, Jinichi Mori, Mutsuko Ohnishi, Larry Wesley Ward, Hitoro Narimatsu, Koichiro Yuji, Masahiro Kami.

**Writing – review & editing:** Naoki Okada, Tetsuya Tanimoto, Asaka Higuchi, Izumi Yoshida, Kazuhiro Kosugi, Yuto Maeda, Yoshitaka Nishikawa, Akihiko Ozaki, Kenji Tsuda, Jinichi Mori, Mutsuko Ohnishi, Larry Wesley Ward, Hitoro Narimatsu, Koichiro Yuji, Masahiro Kami.

## Supplementary Material

Supplemental Digital Content
