# Supplementary material for: A model-based estimation of inter-prefectural migration of physicians within Japan and associated factors: A 20-year retrospective study
Source: Medicine (Baltimore). 2018 Jun 1;97(22):e10878. doi: 10.1097/MD.0000000000010878 (PMC6392712; doi:10.1097/MD.0000000000010878)
Supplement: Supplemental Digital Content [file medi-97-e10878-s001.docx]

| **Table, Supplemental content. Characteristics of the 47 prefectures** | | | | | | | | | | | |
| --- | --- | --- | --- | --- | --- | --- | --- | --- | --- | --- | --- |
| Prefectures | Inflow ratio, %* | The annual number of newly licensed physicians | The increased number of physicians | The number of practicing PPR in 2014 | The annual number of newly licensed PPR | Physician's average age | Female physicians’ ratio, % | Population density in inhabitable land areas, persons per km^2^ | Average salary of the general population, JPY Million | Unemployment ratio of the general population, % | Aged population ratio, |
| Hokkaido | -4.3 | 298.0 | 2738 | 240.5 | 5.3 | 50.7 | 14.6 | 248 | 2.82 | 3.4 | 26 |
| Aomori | -53.1 | 100.0 | 304 | 203 | 6.9 | 52.1 | 15.4 | 424.7 | 2.46 | 4.5 | 27 |
| Iwate | -43.6 | 81.0 | 290 | 204.2 | 5.8 | 52 | 14.9 | 360.1 | 2.57 | 2.9 | 27.9 |
| Miyagi | 31.2 | 99.1 | 1411 | 232.3 | 4.2 | 50 | 17.3 | 746.6 | 2.98 | 3.7 | 22.9 |
| Akita | -54.2 | 99.4 | 357 | 227.1 | 8.6 | 50.2 | 16.9 | 340 | 2.58 | 3.6 | 30.7 |
| Yamagata | -44.7 | 96.9 | 474 | 230.4 | 7.9 | 51.4 | 16.8 | 409.4 | 2.65 | 2.7 | 28.3 |
| Fukushima | -17.3 | 77.7 | 355 | 196.9 | 3.7 | 52.7 | 14.5 | 479.8 | 2.91 | 3.0 | 26.1 |
| Ibaraki | 34.0 | 99.2 | 1535 | 177.7 | 3.3 | 49.7 | 20.1 | 745.8 | 3.25 | 3.3 | 23.8 |
| Tochigi | 4.4 | 99.2 | 1082 | 223.3 | 5.0 | 48.9 | 19.6 | 872.7 | 3.24 | 3.2 | 23.2 |
| Gunma | -1.4 | 103.7 | 1031 | 228.2 | 5.1 | 51.2 | 18.8 | 673.4 | 3.09 | 2.8 | 24.9 |
| Saitama | 223.7 | 102.3 | 4266 | 158.9 | 1.5 | 50.7 | 19.8 | 2795 | 3.26 | 3.2 | 22 |
| Chiba | 245.4 | 98.6 | 4473 | 189.4 | 1.6 | 48.9 | 20.0 | 1760.1 | 3.31 | 3.0 | 23.2 |
| Tokyo | -13.3 | 1285.5 | 13471 | 323.4 | 10.4 | 47.3 | 28.4 | 9460.6 | 4.10 | 3.6 | 21.3 |
| Kanagawa | 35.5 | 375.8 | 6300 | 209.3 | 4.3 | 48.1 | 24.0 | 6167.2 | 3.71 | 3.3 | 21.5 |
| Niigata | -6.3 | 99.0 | 771 | 200.9 | 4.1 | 52.2 | 16.6 | 527.2 | 2.86 | 2.8 | 27.2 |
| Toyama | -39.1 | 92.3 | 499 | 248.2 | 8.3 | 51.8 | 17.6 | 590.1 | 3.03 | 2.3 | 27.6 |
| Ishikawa | -68.2 | 194.1 | 489 | 285.7 | 16.5 | 49.7 | 17.4 | 842.5 | 3.03 | 2.3 | 25 |
| Fukui | -51.7 | 98.3 | 513 | 250.9 | 12.0 | 49.4 | 17.7 | 750.7 | 2.98 | 1.8 | 26 |
| Yamanashi | -52.9 | 95.6 | 465 | 230.2 | 10.9 | 50.3 | 16.8 | 906.3 | 3.13 | 2.8 | 25.6 |
| Nagano | 19.5 | 97.9 | 1293 | 226.9 | 4.5 | 51.4 | 17.5 | 649.6 | 3.05 | 2.7 | 27.4 |
| Gifu | 33.5 | 79.8 | 1214 | 208.8 | 3.8 | 50.7 | 17.9 | 945.7 | 3.05 | 2.3 | 25.2 |
| Shizuoka | 71.7 | 99.4 | 1765 | 201.5 | 2.6 | 50 | 16.6 | 1367.4 | 3.26 | 2.7 | 24.9 |
| Aichi | 7.7 | 380.2 | 4810 | 213.6 | 5.3 | 48.7 | 21.7 | 2490.8 | 3.52 | 2.5 | 21.4 |
| Mie | -9.9 | 99.4 | 928 | 216 | 5.4 | 51.5 | 15.7 | 914.5 | 3.30 | 2.2 | 25.3 |
| Shiga | -14.5 | 98.0 | 1007 | 222.4 | 7.2 | 48.6 | 19.4 | 907.4 | 3.28 | 2.2 | 21.6 |
| Kyoto | -2.7 | 201.6 | 2072 | 326.3 | 7.6 | 48.7 | 22.0 | 1088.1 | 3.31 | 3.2 | 24.7 |
| Osaka | 18.9 | 479.2 | 6025 | 274.6 | 5.4 | 49.5 | 21.2 | 2239.1 | 3.52 | 4.2 | 23.7 |
| Hyogo | 68.4 | 196.9 | 3729 | 242.9 | 3.6 | 50.4 | 19.7 | 6728.7 | 3.19 | 3.8 | 24.3 |
| Nara | -19.3 | 96.8 | 852 | 232.6 | 6.8 | 50.3 | 18.6 | 1645.1 | 3.20 | 3.2 | 25.5 |
| Wakayama | 1.2 | 61.4 | 602 | 287.4 | 5.9 | 50.9 | 18.2 | 2013.6 | 2.91 | 2.5 | 28.4 |
| Tottori | -55.5 | 81.4 | 314 | 311 | 13.4 | 52.1 | 17.2 | 646.4 | 2.74 | 2.7 | 27.2 |
| Shimane | -60.8 | 96.0 | 292 | 279.3 | 12.8 | 50.7 | 19.6 | 556.9 | 2.77 | 2.6 | 30 |
| Okayama | -33.7 | 197.4 | 1355 | 299.4 | 10.1 | 49.8 | 19.4 | 873.4 | 3.03 | 3.0 | 26.2 |
| Hiroshima | 58.2 | 99.1 | 1449 | 263.1 | 3.4 | 51.5 | 18.6 | 1249 | 3.15 | 2.9 | 25.3 |
| Yamaguchi | -31.4 | 96.9 | 459 | 257 | 6.4 | 52.7 | 15.5 | 845.8 | 2.96 | 2.8 | 29.2 |
| Tokushima | -48.2 | 93.1 | 393 | 322.4 | 11.5 | 52 | 22.8 | 767 | 2.85 | 3.0 | 28 |
| Kagawa | -38.9 | 93.9 | 518 | 281.5 | 9.3 | 51.1 | 20.3 | 993 | 2.95 | 2.9 | 27.1 |
| Ehime | -24.5 | 95.2 | 585 | 263.7 | 6.5 | 51.6 | 16.4 | 858.7 | 2.84 | 2.6 | 27.8 |
| Kochi | -56.1 | 93.0 | 281 | 302.4 | 11.7 | 51.4 | 20.1 | 658.7 | 2.69 | 3.0 | 30.1 |
| Fukuoka | 22.8 | 303.0 | 4051 | 307.6 | 6.0 | 49.2 | 19.3 | 1828.6 | 3.04 | 4.1 | 23.3 |
| Saga | -42.0 | 93.7 | 584 | 277.7 | 10.8 | 49.3 | 20.6 | 637.5 | 2.67 | 3.2 | 25.3 |
| Nagasaki | -18.7 | 101.1 | 684 | 300.9 | 6.8 | 52.2 | 17.2 | 873.2 | 2.66 | 3.1 | 27 |
| Kumamoto | 3.2 | 100.5 | 911 | 287.4 | 5.4 | 51.7 | 17.1 | 665.2 | 2.74 | 3.4 | 26.5 |
| Oita | -20.9 | 94.0 | 774 | 271.3 | 7.7 | 51.6 | 17.3 | 685.4 | 2.81 | 2.9 | 27.6 |
| Miyazaki | -35.8 | 97.1 | 625 | 245.1 | 8.4 | 51.4 | 17.5 | 615.1 | 2.59 | 3.2 | 26.7 |
| Kagoshima | -8.6 | 97.5 | 798 | 257.8 | 5.5 | 51.9 | 16.7 | 521.7 | 2.71 | 3.5 | 27 |
| Okinawa | 11.6 | 98.0 | 1492 | 250 | 7.3 | 48.2 | 19.4 | 1193 | 2.46 | 5.1 | 17.7 |

Table illustrates the characteristics of the 47 prefectures.

Abbreviations: PPR, the number of physicians per 100,000 population (physician-to-population ratio).

*Negative values of inflow ratios indicate outflow ratios.
